# Equine ICSI: an update on semen perspective

**DOI:** 10.1590/1984-3143-AR2024-0015

**Published:** 2024-11-22

**Authors:** Rodrigo Arruda de Oliveira, Maria Augusta Alonso, Juliana Schleich Fonte, Claudia Barbosa Fernandes

**Affiliations:** a Laboratório de Reprodução Animal, Faculdade de Agronomia e Medicina Veterinária, Universidade de Brasília, Brasília, DF, Brasil; b Departamento de Reprodução Animal, Faculdade de Medicina Veterinária e Zootecnia, Universidade de São Paulo, São Paulo, SP, Brasil

**Keywords:** cut straw, fertilization, frozen semen, stallion, swim up

## Abstract

Intracytoplasmic Sperm Injection (ICSI) has increased usage in cases of stallion fertility issues, particularly for older stallions, those with reduced sperm numbers or quality, or stallions that have passed away, and only a limited amount of frozen semen is available. By manipulating the frozen semen through thawing, diluting, and refreezing or by cutting the straw under liquid nitrogen, the supply of semen for ICSI can be extended. While ICSI requires a minimal number of spermatozoa per procedure, it is important to consider sperm quality as a crucial factor affecting fertilization and embryo development. Although it is possible to produce healthy embryos and offspring from low quality sperm samples, it is preferable to process and select morphologically and functionally superior sperm to maximize the chances of successful fertilization and embryo development. Several techniques are available for selecting the spermatozoa for ICSI, such as swim-up, washing, density gradient centrifugation, microfluidic sorting, and some combinations. In this review, we will focus on semen type, handling, recent breakthroughs, stallion effects on ICSI efficiency and the prospects of this technology within the equine industry.

## Introduction

The field of assisted reproductive technologies (ARTs) has witnessed remarkable progress and acceptance over recent decades, from the first reports since the early 2000 (Hatzel and Stokes, 2021) revolutionizing our ability to overcome infertility challenges in both humans and animals.

In equine reproduction, one groundbreaking method is Intracytoplasmic Sperm Injection (ICSI), a powerful tool that has demonstrated immense potential in equine breeding programs, both for mares and stallions, especially overcoming or reducing the impacts of acquired subfertility and enabling the production of viable embryos from mares and stallions that are not able to produce embryos via conventional AI (artificial insemination) and ET (embryo transfer) (Morris, 2018; Hinrichs, 2018; Lazzari et al., 2020; Hatzel and Stokes, 2021; Claes and Stout, 2022; Ramírez-Agámez et al., 2023). Moreover, the use of IVP (in vitro production) has been increasing for fertile animals such as Warmblood mares, as it offers a decrease in reproductive management and increase in overall efficiency (more embryos per cycle than conventional ET) (Stout and Griffiths, 2021; Claes and Stout, 2022).

One of the most common uses of ICSI is for those stallions that have limited supply of semen (stallions that died or became infertile), these situations are unsuitable for a conventional embryo transfer program, and OPU-ICSI becomes the technique of choice (Hinrichs, 2005; Stout, 2020; Ramírez-Agámez et al., 2023).

All semen types, including fresh, cooled-transported, frozen, refrozen, epididymal, and even lyophilized, recently sexed-sorted semen, all are viable options for utilization in the ICSI procedure (McCue, 2021) and some studies have shown similar results regardless of the type of semen used (Ramírez-Agámez et al., 2023) while others showed a better outcome using cooled versus frozen semen (Campos-Chillon and Altermatt, 2023).

Frozen semen represents the leading choice for ICSI because it is more convenient for storage and transport than fresh and cooled semen, and it is available in various forms, including conventional cryopreserved dose (typically containing 100-200 million sperm per straw), ICSI doses (containing approximately 1 million sperm per straw), and refrozen ICSI straws (Ramírez-Agámez et al., 2023).

Several techniques are available for selecting the spermatozoa for ICSI, such as swim-up, washing, density gradient centrifugation, microfluidic sorting, and some combinations. Nevertheless, it is essential to highlight that the success of the technique is related to the adequate processing of semen, a critical factor emphasized by McCue (2021). It should also be emphasized that, although stallion factors appear to cause less variation than the mare, male effects are described in the literature and should be considered (Cuervo-Arango et al., 2019).

In this review, we will focus on semen type, handling, recent breakthroughs, stallion effects on ICSI efficiency and the prospects of this technology within the equine industry.

## Sperm type

### Fresh

Although fresh semen is widely used in assisted reproduction techniques in vivo, the focus of commercial ICSI has been on the use of cryopreserved semen, since a semen straw can be reused several times. Moreover, it is more convenient for sperm storage and transport compared to fresh or cooled semen. Studies have indicated that the production of equine embryos by ICSI is similar when using fresh, cooled, or frozen/thawed sperm (Choi et al., 2002, 2004, 2016). Therefore, there are few published works using fresh semen in ICSI. One of them is the publication by Choi et al. (2002), which evaluated the development of equine oocytes in vitro and in vivo after ICSI with either fresh or frozen-thawed spermatozoa and did not find a difference neither for fertilization rate (pronucleus formation and cleavage) at 20 h after injection of spermatozoa nor for cleavage rate or average number of nuclei at 96 h between equine oocytes, concluding that the injection of frozen-thawed equine spermatozoa results in similar embryo development to that obtained with fresh equine spermatozoa. Amer and Fakhry (2021) analyzed 1189 publications, for human ICSI, in a systematic review concluded the use of fresh and frozen sperm showed comparable fertilization and clinical pregnancy rates.

### Cooled

Cooled semen can be used for ICSI, however, collection of the stallion must be scheduled in advance at least one day before the procedure, to ensure that the semen is transported to the laboratory in time, avoiding the risk of the semen delay for the procedure. Semen dose does not need to be at the conventional concentration and total sperm number, as only one sperm per oocyte is needed. Furthermore, if it arrives the day before ICSI, it can be kept refrigerated in its own Styrofoam packaging or in the refrigerator overnight (Rader et al., 2016).

### Frozen

Sixty-seven years have passed since the first foal was born from cryopreserved semen (Barker and Gandier, 1957). Considering that, significant advances have been observed using ART in vivo. However, pregnancy rates remain inconsistent and subject to numerous variables.

The use of frozen semen generated an expansion perspective for equine breeding by enabling the preservation of this material for an indefinite period and allowing worldwide distribution. It maximizes the use of stallions with superior genetic merit and reduces animal transportation costs, as well as diseases. Geographical barriers can be abolished, and there is the possibility of using cryopreserved semen from stallions that are in competition or recovering from pathologies that prevent them from breeding, or even from stallions that have already died (Miller, 2008).

From this perspective, the use of frozen semen for ICSI is commercially interesting as straws with concentrations lower than conventional (100-200) can be frozen and even conventional straws can be used more than once. Even stallions with ejaculatory and osteoarticular disorders can undergo pharmacological ejaculation, and this ejaculate can be cryopreserved for use in ICSI and consequently obtain more pregnancies, maximizing the genetic potential of this stallion.

The use of different semen extenders for cryopreservation, with varying compositions and glycerol percentages, has also been under discussion, as they yield different fertilization rates (Roasa et al., 2007; Cook et al., 2020; Ramírez-Agámez et al., 2023). Although the use of refrozen semen is an alternative for ICSI, research indicates low cleavage and blastocyst rates, due to a reduction in sperm quality (Claes and Stout, 2022).

As known, the semen cryopreservation process can lead to a series of injuries to spermatozoa, such as damage to the plasma membrane, acrosome, and generation of reactive oxygen species (Oliveira et al., 2013; Sieme et al., 2015). It was recently discovered that cryopreservation of equine spermatozoa reduces plasma membrane integrity and phospholipase C zeta 1 (PLCZ1) content, which is associated with oocyte activation (Gonzalez-Castro et al., 2024). During fertilization, the sperm delivers oocyte-activating factors into the ooplasm, inducing oocyte activation and, consequently, cleavage and embryo development (Dozortsev et al., 1997). PLCZ1 is considered the main sperm-borne oocyte activation factor in several mammalian species (Sato et al., 2013; Villaverde et al., 2013; Schrimpf et al., 2014; Amdani et al., 2016; Nozawa et al., 2018). A high content and proper localization of PLCZ1 in human and equine spermatozoa are positively correlated with cleavage rates and ICSI outcomes (Yelumalai et al., 2005; Gonzalez-Castro et al., 2019), supporting the role of PLCZ1 in oocyte activation. Subsequently, frozen-thawed samples exhibiting higher proportions of motile spermatozoa can be further sorted by swim-up, density gradient centrifugation, or microfluidic sorting, facilitating the selection of individual spermatozoa for ICSI, which potentially have a higher PLCZ1 content.

### Frozen epididymal sperm

Considered the last resource to cryopreserve a stallion’s semen, it is an important ART, as it can be carried out if the testicles are removed immediately after the animal’s death, they can be cooled-stored at 5^o^C resulting in good post-thawing rates of the sperm present in the tail of the epididymis if this material reaches the laboratory within 24 hours. Freezing the semen from the epididymis of stallions has become an important tool for preserving the genetics of these animals when they are castrated, or when they die, whether due to illness or euthanasia. Unpublished data from Colorado State University during the 2006/2007 breeding season, suggested that more oocytes were cleaved with thawed epididymal sperm than with frozen-thawed sperm from conventional semen. However, they demonstrated that the rate of early embryonic death is higher in the epididymal sperm group (Bruemmer, 2011). Another study using semen from the epididymis showed that cleavage and blastocyst rates are similar compared to conventionally collected semen, provided that the initial quality of the frozen semen is suitable for the technique (Ramírez-Agámez et al., 2023).

### Sexed-sorted semen

Sexed semen can also be used in the ICSI technique, however a difference in cleavage rates using sexed and unsexed semen is noted (Galli et al., 2008). The cleavage of unsexed semen was significantly greater compared to sexed semen, consequently reflecting the number of blastocysts produced, also shown by Colleoni et al. (2008) and Carnevale et al. (2008). In contrast, Dini et al. (2023) compared the use of sexed and unsexed semen and observed no significant differences in cleavage rates and blastocyst production between the groups, although it is a preliminary study.

## Sperm processing techniques

Despite the numerous benefits of ICSI, is important to note that the manual selection of a sperm for ICSI bypasses the natural selection of viable spermatozoa that would naturally occur in the female reproductive tract, and to a lesser extent during conventional in vitro fertilization (IVF) procedures. Consequently, the absence of natural sperm selection may represent a barrier to optimal fertilization and development. With ICSI, it is possible to select a visually normal spermatozoa possessing damaged DNA or internal structure, which could lead to abnormal embryo development or miscarriage as observed in humans. Therefore, the use of artificial selection techniques to select for the most competent spermatozoa in a sample is a critical step in the optimization of ICSI outcomes (Orsolini et al., 2021).

In vivo, it is believed that sperm are naturally “selected” as they navigate through the female reproductive tract, resulting in only the most competent spermatozoa reaching the oviduct and ultimately fertilizing an ovum (Sakkas et al., 2015). However, these natural sorting procedures are bypassed during IVP, which could contribute to the suboptimal outcome of these techniques.

Although it is possible to produce healthy embryos and offspring from low quality sperm samples, it is preferable to process and select morphologically and functionally superior sperm to maximize the chances of successful fertilization and embryo development.

For frozen semen, both whole straw and a cut straw could be used. In commercial ICSI programs, a common practice involves cutting a small portion of a frozen semen straw while still submerged in liquid nitrogen. It is called "ICSI-cuts" with 6 to 10 cuts produced per straw. A small piece (<5mm) of the frozen straw is cut, under liquid nitrogen, and placed in a tube containing sperm media and placed on an incubator (38.2 ^o^C), where it remains for 15 minutes, and 0.5 mL of the supernatant is placed in a 15 mL conical tube. The tube is then centrifuged at 300-350 x g for 3-5 minutes to form a pellet in the bottom. Further, semen is incubated for 5-20 minutes, allowing sperm to swim up out of the pellet to the supernatant. A small volume of the supernatant is used for identifying the spermatozoa for ICSI (Lazzari et al., 2020; Rader et al., 2016; McCue, 2021).

Each of these cuts can be thawed separately for individual ICSI procedures. Moreover, depending on the post-thaw motility of a frozen ejaculate, straws of frozen semen can be thawed, diluted to about 1 million sperm per mL, and refrozen, potentially leading to hundreds of ICSI doses (Rader et al., 2016). The remainder of the straw is placed back into a labeled goblet, attached to a labeled cane, and replaced into the storage tank of liquid nitrogen. Some facilities place an inverted goblet to prevent floating out of the remainder of the straw during storage (McCue, 2021). This strategic approach is essential, particularly in cases where the semen supply is limited (McCue, 2021).

One of the advantages of using spermatozoa in ICSI is that, unlike IVF, they do not need to be pre-capacitated with calcium ionophore (Leemans et al., 2016). According to the authors, they hypothesize that one or more oviduct-derived factor(s) is indispensable to adequate triggering of capacitation to enable stallion cells to penetrate an oocyte. The key capacitating factor composition of oviduct fluid is usually mimicked in vitro to establish an efficient capacitation/fertilization medium. The effect of increased local pH on capacitation strongly suggests that equine sperm capacitation/fertilization media require a higher pH than for other mammalian species. However, even these capacitating conditions do not induce the acrosome reaction and allow successful equine IVF.

### Swim-up

According to Rader et al. (2016), the swim-up technique is the most used. This technique consists of adding 100 to 200 µL of chilled or thawed semen in Eppendorf, and medium is added above. It remains for 20 minutes at 38.2 ^o^C, the upper part of the sample (about 0.6 mL) is removed with a pipette and placed in another Eppendorf. This suspension is then centrifuged for 3 minutes at 327 g. After centrifugation, the pellet is resuspended in the medium containing 1 mL and centrifuged again. The remaining supernatant is removed, and the sample pellet is resuspended in the remaining medium.

### Density gradient centrifugation

Gradient centrifugation is the superposition of a semen layer on top of one or two layers of colloid in a centrifuge tube (Henkel and Schill, 2003), which is then centrifuged at 300-600g for 15 to 30 minutes, enabling separation of greater quality sperm (Orsolini et al., 2021). When a double centrifugation layer is used, the less dense top layer will retain the macromolecules, leukocytes and cell debris (De Martin et al., 2019), sperm easily pass through this and the second colloid layer, which is denser and will retain defective sperm. Morphologically normal sperm are denser and will penetrate the silica layer and form a pellet at the bottom of the tube (Macpherson et al., 2002; Edmond et al., 2008).

### Density gradient centrifugation plus swim up

It is also possible to use the combination between density gradient centrifugation and swim-up. After formation of the sperm pellet with viable sperm, swim up technique is used to select the sperm. It has been demonstrated that using both techniques combined improves the selection of sperm, which have greater motility and morphology compared to using only one of them (Ng et al., 1992; Yamanaka et al., 2016). Another advantage of combining both methods is to remove pathogens from contaminated semen with equine viral arteritis (Morrell and Geraghty, 2006).

### Microfluidic sorting

Although sperm selection techniques such as swim-up and density gradients have been widely used in the past, research has shown promising results with the use of microfluidic devices. This technique, which has been extensively utilized in human IVF recently, does not require centrifugation—a process that can potentially cause damage to spermatozoa. Additionally, it results in lower generation of reactive oxygen species and less DNA damage (Matsuura et al., 2013; Asghar et al., 2014; Nosrati et al., 2014; Shirota et al., 2016; Gonzalez-Castro and Carnevale, 2019). A study was conducted to compare Microfluidic sorting (MF) with single-layer colloidal centrifugation (SLC) and swim-up (SU), showing similar motility, viability and membrane integrity for MF and SLC, but greater morphology and DNA integrity for MF than SLC. And they showed that selection using MF did produce similar cleavage and blastocyst rates than SLC and can be an alternative method for ICSI semen selection (Gonzalez-Castro et al., 2018; Gonzalez-Castro and Carnevale, 2019).

## Stallion effects on ICSI efficiency

Although the mare identity and number of oocytes recovered had greater effect on overall ICSI efficiency compared to stallion (Fonte et al., 2024), this factor should be discussed (Claes and Stout, 2022) and was already demonstrated by some authors (Galli et al., 2016; Cuervo-Arango et al., 2019). Stallion breed, batch of semen are some factors that showed effect on blastocyst production, but interestingly just a few stallions were unable to produce blastocyst after ICSI (Galli et al., 2016; Cuervo-Arango et al., 2019). As expected, a relationship between in vivo and in vitro fertilizing potential was described (Colleoni et al., 2012; Galli et al., 2016).

## Conclusions

The quality of the semen sample is pivotal for the success of Intracytoplasmic Sperm Injection (ICSI). Various factors such as breed, in vivo fertility, sperm preparation technique, and specific batch influence the results. Identifying these factors in a clinical environment is complex due to the added variability from both stallion and mare.

ICSI can be effectively performed using fresh, cooled, or frozen semen. Various processing methods are viable, and they exhibit no significant differences in outcomes. This innovative approach enables the utilization of semen from stallions with limited availability, including those deceased or with subfertility or infertility, who are unlikely to produce offspring through conventional in vivo methods such as artificial insemination and fresh embryo.
